# Health-related quality of life determinants among Rwandan women after delivery: does antenatal care utilization matter? A cross-sectional study

**DOI:** 10.1186/s41043-018-0142-4

**Published:** 2018-04-27

**Authors:** Regis Hitimana, Lars Lindholm, Gunilla Krantz, Manasse Nzayirambaho, Jeanine Condo, Jean Paul Semasaka Sengoma, Anni-Maria Pulkki-Brännström

**Affiliations:** 1 School of Public Health, College of Medicine and Health Sciences, University of Rwanda, Kigali, Rwanda; 2 Epidemiology and Global Health Unit, Department of Public Health and Clinical Medicine, Umeå University, Umeå, Sweden; 3 Section of Epidemiology and Social Medicine (EPSO), Department of Public Health and Community Medicine, The Sahlgrenska Academy, Gothenburg University, Gothenburg, Sweden; 4 Rwanda Biomedical Centre, Kigali, Rwanda; 5 Department of Clinical Sciences, Obstetrics and Gynecology, Umeå University, Umeå, Sweden

**Keywords:** Antenatal care, Health-related quality of life, HRQoL, MaTHeR, Social support, Wealth, Postnatal women

## Abstract

**Background:**

Despite the widespread use of antenatal care (ANC), its effectiveness in low-resource settings remains unclear. In this study, self-reported health-related quality of life (HRQoL) was used as an alternative to other maternal health measures previously used to measure the effectiveness of antenatal care.

The main objective of this study was to determine whether adequate antenatal care utilization is positively associated with women’s HRQoL. Furthermore, the associations between the HRQoL during the first year (1–13 months) after delivery and socio-economic and demographic factors were explored in Rwanda.

**Methods:**

In 2014, we performed a cross-sectional population-based survey involving 922 women who gave birth 1–13 months prior to the data collection. The study population was randomly selected from two provinces in Rwanda, and a structured questionnaire was used.

HRQoL was measured using the EQ-5D-3L and a visual analogue scale (VAS). The average HRQoL scores were computed by demographic and socio-economic characteristics. The effect of adequate antenatal care utilization on HRQoL was tested by performing two multivariable linear regression models with the EQ-5D and EQ-VAS scores as the outcomes and ANC utilization and socio-economic and demographic variables as the predictors.

**Results:**

Adequate ANC utilization affected women’s HRQoL when the outcome was measured using the EQ-VAS. Social support and living in a wealthy household were associated with a better HRQoL using both the EQ-VAS and EQ-5D. Cohabitating, and single/unmarried women exhibited significantly lower HRQoL scores than did married women in the EQ-VAS model, and women living in urban areas exhibited lower HRQoL scores than women living in rural areas in the ED-5D model. The effect of education on HRQoL was statistically significant using the EQ-VAS but was inconsistent across the educational categories. The women’s age and the age of their last child were not associated with their HRQoL.

**Conclusions:**

ANC attendance of at least four visits should be further promoted and used in low-income settings. Strategies to improve families’ socio-economic conditions and promote social networks among women, particularly women at the reproductive age, are needed.

## Background

Maternal mortality remains a principal measure of maternal health despite the widespread recognition that the maternal death rate is only a single indicator of ill maternal health [1, 2]. Recent studies have quantified problems, such as mental distress, genital infections, breast problems [3, 4], physical complaints, pain, sleep problems [4], urinary incontinence, and anemia [5], occurring during the postpartum period. However, the available data do not fully describe maternal health according to the definition of health as “physical, mental and social well-being and not merely the absence of disease or infirmity” [6]. Women’s health-related quality of life (HRQoL) during the postpartum period is affected by their living conditions [7–12], reproductive history [7, 13, 14], and exposure to and use of reproductive health and antenatal care services [5, 15]. Maternal complications can have long-lasting consequences that impact the lives of women and their families for a long period [16].

The umbrella term antenatal care describes medical and social practices performed during pregnancy [17]. Despite the widespread use of antenatal care, its effectiveness in low-resource settings remains unclear [18, 19]. According to various observational studies, antenatal care has positive effects, such as lower maternal and perinatal mortality and better pregnancy outcomes [20]. Evidence of the effectiveness of antenatal care is necessary for decision-makers to establish adequate policies and strategies and allocate proper resources for their implementation. The self-reported HRQoL is an appropriate outcome measure in evaluations of maternal health interventions because this measure is often used in health economic evaluations [21] and can capture health events that are rarely fatal [22].

In this study, we used data obtained from a cross-sectional population-based survey in Rwanda to investigate women’s HRQoL during the first year after delivery. Our main objective was to determine whether adequate antenatal care utilization, which is a binary variable defined according to the number of visits and timing of the first visit, is positively associated with women’s HRQoL. Furthermore, we explored the association between HRQoL and socio-economic and demographic factors. To the best of our knowledge, this study is the first investigation of women’s self-reported HRQoL after delivery in Rwanda. Our study contributes to the literature regarding the burden of maternal morbidity and, ultimately, to governmental health and social policies designed to improve women’s well-being.

## Methods

### Study design and setting

This cross-sectional population-based survey involved women who gave birth between 1 and 13 months prior to the data collection.

The study population was randomly selected from all districts in Kigali City and the Northern Province. Kigali City has three districts, and the Northern Province has five districts. Kigali City has a population of 1,135,428, and most residents live in areas with urban characteristics (i.e., housing, economic activities, and access to infrastructures), while the Northern Province has 1,729,927 inhabitants living mainly in rural areas [23]. The total population in the selected provinces represents 27.2% of the country’s total population [23]. Kigali City and the Northern Province were chosen not only because of logistical reasons but also because these areas are considered to reflect the situation in other provinces based on various surveys conducted in Rwanda showing no major regional differences in the reproductive health indicators [24].

### Participant selection

The sample size was calculated based on the estimated prevalence of hypertension as a pregnancy complication (*p* = 10%) [25], a precision rate of 5%, and projected non-response rates of 10 and 5% to account for the design effect.

Three-stage sampling was conducted to identify the households to include in the study. First, in eight districts (three in Kigali City and five in Northern Province), 48 primary sampling units, i.e., villages, which represent the lowest administrative unit in Rwanda, were randomly selected from a total of 3918 villages in the two provinces, corresponding to 1.2% of the total number of villages [26]. Twenty percent of the villages were selected from urban areas, and 80% of the villages were selected from rural areas to reflect the rural-urban proportions in Rwanda. Second, the number of households selected in each village was decided according to the proportion to the total number of households in each village. In each village in Rwanda, community health workers maintain records of women expecting to deliver and women with newborns and infants less than 1 year of age. Third, these records were used to randomly select the households to be visited, and the list was reduced to only include households with a woman who fulfilled the main inclusion criterion, i.e., delivery within 1 to 13 months before the survey.

If a village did not have the desired number of households fulfilling the inclusion criterion, the remaining households were selected from a neighboring village. In total, 922 women were selected and invited to participate. All women agreed to participate.

### Data collection procedure

A questionnaire comprising socio-economic and demographic factors, health conditions, and the use of maternal health services was developed in English and translated into Kinyarwanda by a professional translator with experience in translating medical and public health questionnaires in Rwanda.

The data collection was performed by the University of Rwanda, School of Public Health between July and August of 2014. In addition to four PhD students who were responsible for leading the fieldwork and their five supervisors, 12 female data collectors were hired. The data collectors had previous training in nursing or a related subject (minimum of 6 years of post-primary school) and experience in population-based data collection. The data collectors received a 4-day training, including 1 day of piloting in one village (not included in the sampled area). Data entry was performed by four experienced data clerks trained on using an SPSS data-entry template (SPSS version 22.0) [27] and supervised by a data manager.

### Measures

#### Dependent variable

The dependent variable is the HRQoL, which was measured according to the EQ-5D praxis. The following two methods [28] were used:

(1) The EQ-5D-3L descriptive system (designated EQ-5D) comprises the following five dimensions: mobility, self-care, usual activities, pain or discomfort, and anxiety or depression. The respondents selected among three options (i.e., no problems, some problems, severe problems) in response to each dimension. Each response provides a combination (e.g., 1, 1, 2, 1, and 2) that has a corresponding value, often called a score [28]. Given the lack of an official version of the EQ-5D-3L in any language spoken in Rwanda, we translated the English version of the EQ-5D-3L into Kinyarwanda and obtained retrospective permission to use the translated questionnaire from EuroQol. To calculate the EQ-5D scores, we used the weights used in a population study conducted in the UK [29].

(2) Using the visual analogue scale (designated EQ-VAS), the women were asked to express how good or poor their health was on the day of the interview by indicating a point on a scale from 0 to 100. A score of 100 represented the best imaginable health state, while a score of 0 represented the worst imaginable health state.

#### Independent variables

The main independent variable was adequacy of antenatal care utilization, which is a binary indicator of whether a woman had received antenatal care according to Rwandan guidelines. This variable was constructed using Kessner’s index [30] as a prototype and adapted to the Rwandan antenatal care guidelines. The Rwandan antenatal care policy was developed based on the 2002 WHO guidelines, which suggested four focused antenatal visits for normal pregnancies [31]. However, most Rwandan couples do not adhere to this recommendation. For example, in 2014/15, only 45% of women completed four visits, and the average month of initiation of the first antenatal visit was the fifth month of gestation [24]. The following two categories of this variable were constructed:

1. The women were categorized as having *adequate antenatal care (ANC) utilization* if they had completed at least four ANC visits and the first visit occurred during the first trimester.
2. The women who did not meet the abovementioned criterion were categorized as having *inadequate ANC utilization*.

The women’s socio-economic and demographic characteristics were divided into the following groups: residence was divided into two groups (i.e., rural and urban) according to the location of the village; age was divided into groups in 5-year intervals; educational level was divided into four groups (i.e., some primary school, completed primary school, lower secondary or vocational education, and upper secondary or higher education); and marital status was divided into four groups (i.e., married, cohabitating, separated/divorced/widowed, and unmarried/single).

A household wealth index was constructed by performing principal component analysis and using information regarding housing characteristics (i.e., materials used to construct walls, source of water, type of cooking fuel, and connection to electricity) and ownership of durable assets (i.e., mattress, iron, TV, mobile phone, and computer). The household scores were standardized based on a normal distribution with a mean of zero and a standard deviation of one [32]. The households were ranked according to these scores and divided into the following five equal groups: lowest, second, middle, fourth, and highest wealth quintiles.

The social support variable was constructed from seven questions regarding the respondents’ access to four main types of social support, i.e., emotional, tangible, or instrumental, informational and appraisal support, which are often referenced in the medical literature [33, 34]. The responses were dichotomized, given the values 0 (never) or 1 (sometimes, often or always) and summed for all respondents. Thus, the respondents were assigned social support values ranging between zero and seven. Finally, the values were grouped into the following two categories: poor social support (access to 0–4 types of social support) and good social support (access to 5–7 types of social support).

#### Statistical analysis

The average EQ-5D scores and 95% confidence intervals were calculated in relation to all independent variables. First, a bivariate analysis was performed using independent *t*-tests and one-way analyses of variance to identify the significant differences in the mean HRQoL values among different groups of independent variables at a 0.05 significance level. Second, two multivariable linear regression models were constructed using the EQ-5D scores and EQ-VAS scores as the outcomes and the adequacy of ANC utilization and socio-economic and demographic variables as the predictors. All predictors included in the bivariate analysis were considered in the initial model, except for the variable “Number of children” because this variable had a high proportion of missing values (31.4%), which led to biased model results. A backward stepwise procedure was performed at a *p* value of 0.05 to identify the variables that remained significant in the final model. The co-linearity among the covariates was assessed by performing a variance inflation factor (VIF). None of the covariates presented a VIF value above the maximum acceptable value of 10. The data analysis was performed using STATA 13 [35]. Following Pullenayegum et al. [36], the ordinary least squares with heteroskedasticity-consistent (robust) standard errors method was used as a simple and valid estimation method when many individuals achieved the upper bound of one as in our EQ-5D data and because our sample size can be considered large.

## Results

The socio-economic and demographic characteristics of our study population are described in Table 1. The antenatal care utilization was categorized as inadequate in most (62%) women.

**Table 1:** Distribution of women by socio-economic and demographic categories (*N* = 922)

Variables	Number	Percent	[95% CI]
Residence			
Rural	706	76.7	73.8–79.3
Urban	215	23.3	20.7–26.2
Age groups			
15–20	95	10.3	8.5–12.5
21–25	267	29.0	26.2–32.0
26–30	271	29.5	26.6–32.5
31–35	173	18.8	16.4–21.5
36–40	85	9.2	7.5–11.3
> 40	29	3.1	2.2–4.5
Education level			
Some primary	417	50.2	46.8–53.6
Primary	202	24.3	21.5–27.3
Lower secondary or vocational	112	13.5	11.3–15.9
Upper secondary or higher education	100	12.0	9.9–14.4
Marital status			
Married	482	52.4	49.1–55.6
Cohabitant	292	31.7	28.8–34.8
Separated/divorced/widow	23	2.5	1.6–3.7
Unmarried/single	123	13.4	11.3–15.7
Social support categories			
Poor social support	315	34.6	31.5–37.7
Good social support	596	65.4	62.3–68.4
Number of children			
1 child	41	6.5	4.8–8.7
2 children	227	35.9	32.3–39.8
3 children	148	23.4	20.3–26.9
4 to 5 children	138	21.8	18.8–25.3
≥ 6 children	77	12.2	9.8–15.0
Wealth quintiles			
Lowest	168	20.3	17.7–23.2
Second	167	20.2	17.6–23.1
Middle	192	23.3	20.5–26.3
Fourth	150	18.2	15.7–20.9
Highest	148	17.9	15.4–20.7
Adequacy of ANC utilization			
Inadequate ANC	563	61.5	58.3–64.6
Adequate ANC	352	38.5	35.4–41.6

Seventy-seven percent (706) of the respondents lived in rural areas, and 23% (215) of the respondents lived in urban areas. Eighty-eight percent (806) of the women were 35 years old or younger. Half of the women had not completed primary level education, 24% of the women had completed primary level education, and only 12% of the women had completed upper secondary or university level education. Most women (52%) were legally married, 32% of the women were cohabiting, 13% of the women were unmarried/single, and 2% of the women were separated, divorced, or widowed. Most women (65%) had good social support, while 35% of the women had poor social support. Six percent (41) of the women had one child, 36% (227) of the women had two children, 23% (148) of the women had three children, 22% (138) of the women had four or five children, and 12% (77) of the women had six or more children.

### How are the respondents distributed across the different HRQoL levels?

In general, the women did not report problems with mobility, self-care or usual activities, as follows: 95% (880) of the women reported “no problems in walking about,” 99% (912) of the women reported “no problems with self-care,” and 94% (869) of the women reported “no problems with performing my usual activities” (Table 2). Only 5% (42) of the women reported “some problems in walking about,” 1% (10) of the women reported “some problems washing or dressing myself,” and 5% (49) of the women reported “some problems with performing my usual activities.”

**Table 2:** Prevalence of the dimensions of quality of life (*N* = 922**)**

	Number	Percent
Mobility		
No problems	880	95.4
Some problems	42	4.5
Severe problems	0	0
Self-care		
No problems	912	98.9
Some problems	10	1.1
Severe problems	0	0
Usual activities		
No problems	869	94.4
Some problems	49	5.3
Severe problems	2	0.2
Pain or discomfort		
No problems	770	83.6
Some problems	140	15.2
Severe problems	11	1.2
Anxiety or depression		
No problems	745	80.9
Some problems	152	16.5
Severe problems	24	2.6

Eighty-four percent (770) of the women reported no pain or discomfort, while 15% (140) of the women reported having moderate pain or discomfort, and 1% (11) of the women reported having extreme pain or discomfort. Eighty-one percent (745) of the respondents reported that they were not anxious or depressed, 16% (152) of the respondents reported being moderately anxious or depressed, and 3% (24) of the respondents reported being extremely anxious or depressed (Table 2).

### Average HRQoL values by socio-economic and demographic characteristics

The EQ-5D scores on the five dimensions and visual analogue scale (VAS) responses are displayed in Table 3 by the socio-economic and demographic characteristics of the respondents.

**Table 3:** Average health-related quality of life values by socio-economic and demographic characteristics (*N* = 922)

	Number	EQ-5D score (mean)	[95% CI]	Number	EQ-VAS score (mean)	[95% CI]
Residence						
Rural	705	0.928	0.916–0.940	704	69.1	67.6–70.6
Urban	214	0.903	0.878–0.928	210	71.2	68.8–73.5
Age groups						
15–20	95	*0.930*	*0.899–0.960*	94	69.8	65.5–74.0
21–25	266	*0.933*	*0.916–0.950*	264	69.0	66.5–71.5
26–30	271	*0.908*	*0.885–0.931*	270	69.2	66.8–71.6
31–35	173	*0.932*	*0.912–0.953*	171	70.7	68.1–73.3
36–40	84	*0.933*	*0.895–0.971*	85	70.4	65.9–74.9
> 40	29	*0.839*	*0.736–0.942*	29	67.9	61.4–74.4
Education level						
Some primary	416	0.915	0.898–0.932	*415*	*68.8*	*66.8–70.7*
Primary completed	200	0.943	0.921–0.963	*201*	*67.7*	*65.1–70.3*
Lower secondary or vocational	111	0.918	0.888–0.947	*108*	*71.8*	*68.6–75.1*
Upper secondary or university	100	0.944	0.921–0.967	*99*	*76.2*	*73.1–79.2*
Marital status						
Married	481	*0.936*	*0.922–0.949*	480	*72.6*	*70.9–74.2*
Cohabitant	290	*0.915*	*0.893–0.937*	290	*67.8*	*65.4–70.1*
Separated/divorced/widow	23	*0.839*	*0.754–0.923*	22	*61.1*	*53.1–69.1*
Unmarried/single	124	*0.902*	*0.872–0.932*	121	*63.4*	*59.3–67.6*
Social support categories						
Poor social support	313	*0.883*	*0.859–0.906*	312	*61.9*	*59.5–64.3*
Good social support	595	*0.944*	*0.934–0.955*	592	*73.7*	*72.3–75.1*
Number of children						
1	41	*0.996*	*0.988–1.003*	41	76.1	70.5–81.7
2	228	*0.901*	*0.874–0.926*	225	68.3	65.8–70.8
3	146	*0.942*	*0.921–0.962*	146	71.8	68.6–75.1
4 to 5	137	*0.924*	*0.896–0.953*	137	68.6	65.6–71.6
≥ 6	77	*0.879*	*0.825–0.933*	77	69.3	64.3–74.2
Wealth quintiles						
Lowest	166	0.893	0.858–0.927	167	*63.5*	*60.3–66.8*
Second	167	0.928	0.903–0.952	167	*69.5*	*66.3–72.7*
Middle	191	0.939	0.919–0.959	190	*71.8*	*69.4–74.3*
Fourth	150	0.912	0.887–0.936	148	*69.7*	*67.0–73.2*
Highest	148	0.937	0.910–0.963	146	*76.3*	*73.6–78.8*
Child’s age						
≤ 2 month	128	0.926	0.901–0.951	126	71.5	67.5–75.6
3–4 month	185	0.941	0.923–0.960	185	69.8	66.9–72.6
5–6 month	145	0.908	0.875–0.942	147	69.9	67.2–72.6
7–8 month	132	0.897	0.864–0.931	129	70.1	66.7–73.5
9–10 month	139	0.914	0.884–0.945	137	67.6	64.1–71.1
11–12 month	126	0.931	0.905–0.957	126	68.8	65.2–72.4
> 12 month	60	0.944	0.903–0.984	61	70.1	65.3–74.8
ANC utilization						
Inadequate ANC	561	0.917	0.902–0.931	*558*	*68.3*	*66.6–69.9*
Adequate ANC	351	0.932	0.917–0.948	*349*	*71.8*	*69.8–73.8*

Statistically differences in bivariate analyses are indicated in italics

The median HRQoL score using the EQ-5D was 1.00 (no problems), the median response to the EQ-VAS was 70 (scale 0–100), and the mean values were 0.92 and 69.58, respectively. The minimum and maximum EQ-5D scores were − 0.18 and 1, while the minimum and maximum EQ-VAS scores were 0 and 100, respectively.

No statistically significant difference was observed in the HRQoL between women who had adequate antenatal care attendance and those with inadequate antenatal care attendance using the EQ-5D as the outcome. Nevertheless, using the EQ-VAS, a significant difference of three and a half points on the 100-point scale of HRQoL was observed between the women who had adequate antenatal care attendance and those who had inadequate attendance.

The women older than 40 years had significantly lower HRQoL scores than did the women in the younger age groups using the EQ-5D. The EQ-VAS did not reveal any significant differences among the age groups.

Regarding the educational level, using the EQ-5D, no statistically significant differences were observed among the groups; however, using the EQ-VAS, the respondents who had completed upper secondary level or university level education had significantly higher HRQoL than did the women in all other groups, followed by the group of women who completed lower secondary or vocational training. The women who had not finished primary education and those who had finished primary education exhibited nearly identical scores, and these women had significantly lower HRQoL than the women in the other groups.

Separated, divorced, or widowed women and unmarried or single women had significantly lower HRQoL than married and cohabiting women using both the EQ-5D and EQ-VAS. Although the difference was small using the EQ-5D, this difference was more noticeable using the EQ-VAS. The separated, divorced, or widowed women scored 11.5 points lower than the married women, and the single or unmarried women scored 9.2 points lower than the married women.

Women benefitting from good social support had a significantly higher HRQoL than did women with poor social support using both methods. Regarding wealth, the women in the highest wealth quintile reported significantly higher HRQoL than all other women using the EQ-VAS. However, using the EQ-5D, no significant difference was observed in the HRQoL among the different wealth quintiles. Women with six or more children had a significantly lower HRQoL than did women with fewer children using the EQ-5D. However, the differences were not significant using the EQ-VAS.

Lastly, respondents did not exhibit statistically significant differences in HRQoL between rural and urban respondents nor between different groups of respondents according to the age of their last child.

### Health-related quality of life determinants: a multivariable approach

After eliminating all non-significant (*p* value> 0.05) exposure variables, the final regression models of the two outcome variables, i.e., EQ-5D and EQ-VAS, are presented in Table 4. For the EQ-5D, robust standard errors were used because there was evidence of heteroskedasticity (*χ*^2^ = 164 vs. *χ*^2^ = 8.95 using the EQ-VAS).

**Table 4:** Association between health-related quality of life and antenatal care utilization: regression analysis using the EQ-5D and EQ-VAS

	Model 1 (EQ-5D as the outcome)			Model 2 (EQ-VAS as the outcome)		
	Coef.	*P*	[95% C. I.]	Coef.	*P*	[95% C. I.]
Social support (ref = Poor social support)						
Good social support	0.062	0.000	0.033; 0.091	9.222	0.000	6.264; 12.180
Wealth quintiles (ref = Lowest)						
Second	0.024	0.287	−0.020; 0.069	4.124	0.081	−0.516; 8.766
Middle	0.047	0.016	0.009; 0.086	5.807	0.005	1.781; 9.834
Fourth	0.005	0.813	−0.038; 0.049	4.399	0.063	−0.239; 9.038
Highest	0.059	0.013	0.012; 0.107	9.200	0.000	4.615; 13.785
Place of residence (ref = Rural)						
Urban	−0.044	0.008	−0.077; − 0.011			
Educational level (ref = Some primary)						
Primary completed				−4.260	0.012	−7.583; −0.936
Lower secondary or vocational				−0.266	0.895	−4.216; 3.684
Upper secondary or university				2.205	0.292	−1.901; 6.312
Marital status (ref = Married)						
Cohabitant				−4.164	0.006	−7.150; −1.178
Separated				−5.757	0.263	−15.848; 4.334
Unmarried/single				−7.585	0.001	−12.069; −3.101
ANC utilization (ref = Inadequate ANC attendance)						
Adequate ANC				3.551	0.008	0.924; 6.179

Notes: Using robust standard errors

The adequacy of antenatal care utilization, which was the main independent variable in this analysis, affected the women’s HRQoL when the outcome was measured using the EQ-VAS. The women who had adequate antenatal care utilization had HRQoL scores that were 3.5 points higher on the VAS scale compared to the women with inadequate antenatal care utilization (Table 4).

Regarding the other socio-economic and demographic variables, “social support” and “wealth” were the only variables that remained statistically significant in both regression models, while the other variables, including place of residence, marital status, and educational level, remained in only one model. On one side, the women with good social support scored 9 points higher on their HRQoL using the VAS score than the women with poor social support. Similarly, the women in the highest wealth quintile scored 9 points higher on the HRQoL than the women in the lowest wealth quintile using the EQ-VAS. On the other side, using the EQ-5D, women with good social support scored 0.059 higher than those with poor social support; and women in the highest wealth quintile had 0.062 point higher scores than those in the lowest quintile.

Using the EQ-5D descriptive system, living in urban areas was associated with significantly lower HRQoL than was residing in rural areas. Using the EQ-VAS, cohabitating women and unmarried/single women had lower HRQoL than married women.

## Discussion

This study aimed to (1) assess whether adequate antenatal care utilization is associated with women’s HRQoL and (2) explore socio-economic and demographic factors of women’s HRQoL during the first year (1–13 months) after delivery in Rwanda.

Regarding the first objective, in this study, adequate antenatal care utilization was associated with better HRQoL using the EQ-VAS as the outcome, while no association was observed using the EQ-5D. The finding contributes to the limited literature. Bahrami et al. concluded that women who received antenatal education had higher levels of happiness, satisfaction, and overall QoL and health during the first postpartum year [15]. Similarly, Parker et al. concluded that women who attended more antenatal care visits had better HRQoL during the postpartum period in a Brazilian population [37]. Similarly, antenatal care has positive effects in improving pregnancy outcomes [38] and reducing women’s morbidity [19, 39] and mortality [19]. However, Zanconato et al. argued that the effectiveness of antenatal care in reducing maternal morbidity and mortality is uncertain in low-income countries despite the convincing evidence in high-income countries due to the shortcomings in the provisions and use of the service [40]. Thus, the effectiveness of antenatal care in low-income countries depends on how well this service is provided [41] and the availability of other services, such as obstetric care [42].

Regarding the second objective of this study, according to our results, having good social support positively affects women’s HRQoL during the first postpartum year. Our findings are consistent with those of several other studies investigating social support as a predictor of the HRQoL during the postpartum period [7, 10, 13, 43]. Possible explanations include that poor social support is a general predictor of postpartum depression [44], stressful events, panic disorders, psychological distress [45], and a poor mental health status [46]. Furthermore, of the five EQ-5D domains, anxiety or depression had the highest proportion of women reporting moderate or severe problems. Social support after giving birth is particularly important in the Rwandan culture. Considering the numerous practices by family and friends that comfort women during the postpartum period, new mothers find it challenging to cope with the situation with inadequate social support.

Household wealth was another socio-economic determinant of women’s HRQoL during the postpartum period. Thus, our results add to the vast literature on health inequalities in different contexts. Several previous studies investigating women’s HRQoL during the postpartum period have demonstrated the influence of household wealth or income [7, 8, 11] or other closely related indicators, such as employment and standard of living [47]. In the model using the EQ-VAS as the outcome, marital status was also significantly associated with HRQoL. In this study, higher educational levels were not associated with better HRQoL. This finding is surprising given that other studies have found good education to be a key predictor of HRQoL [7, 11]. The lack of association between the educational level and HRQoL may be partially attributable to the community health program in Rwanda, which has reduced inequalities in the use of services [48]. Other studies have reported findings similar to ours regarding marital status [8, 37]. However, notably, marital status is not the same among women in different societies [49]. In the Rwandan context, being a single woman is often associated with stigma and has been associated with poor utilization of maternal health services such as antenatal care [50].

In this study, rural women had better HRQoL than did urban women. No consensus exists in the literature regarding the rural-urban differences in HRQoL. Several scholars have argued that rural populations are less likely to be educated and have limited access to infrastructures; therefore, these populations are likely to have lower HRQoL [51]. Other scholars argue that life in urban areas is more stressful, translating to a higher prevalence of anxiety, depression, or other mental health problems [9, 52]. This hypothesis may be supported by our findings because anxiety or depression was the dimension with the widest gap between the rural and urban women as follows: on average, 85% of the women living in rural areas reported not having any anxiety or depression problems, compared with 70% of the women living in urban areas.

Regarding the number of children, although this variable was excluded from the regression analysis, according to the bivariate analysis, women with six or more children had a significantly lower HRQoL than women with fewer children using the EQ-5D. Akýn et al. [7] also established a negative association between the number of children and women’s HRQoL during 12 months postpartum. For women in settings such as Rwanda, where many individuals are rural farmers who must perform farming activities along with caring for their families, partner support is limited, and having many children may be particularly stressful.

The association of the maternal age and age of the baby with HRQoL has been previously reported [7, 14, 53] but was not supported in this study.

### Methodological limitations

A major strength of our study is that we present data on women’s HRQoL collected through a population-based survey. In low-income countries, such as Rwanda, most existing evidence on postpartum morbidity is health facility based; however, very few women with postpartum health problems seek health care [3, 54].

A limitation shared by this study and other studies in the literature is that the quality and content of the antenatal care service were not explored. ANC quality is potentially a determinant of postpartum HRQoL, and thus, there is a risk of omitted variable bias. Including the ANC quality in the analysis may have also enabled a better disentanglement of the role of adequate timing and number of visits. In the case of poor quality or when the content does not meet the required standard, ANC would unlikely have much impact on HRQoL. This limitation has been noted in other studies that assessed the effectiveness of antenatal care [18].

The difference between the two outcome measures (i.e., EQ-5D and EQ-VAS) is important. In our data, more variation was observed in the HRQoL using the EQ-VAS compared to that using the EQ-5D. The social support and wealth results were similar using both methods, but using the EQ-5D, the magnitude of the estimates was small, and the model only explained a small proportion of the overall variation. Lastly the cross-sectional nature of this study does not allow for inferences regarding causal-effect relationships.

## Conclusions

Based on the findings in this study, the adequacy of antenatal care utilization, which is defined as at least four ANC visits and the first visit occurring during the first trimester, is associated with women’s HRQoL during the first postpartum year. Furthermore, having good social support and living in a wealthy household are associated with higher HRQoL. These results support the current call for decision-makers to consider the role of social determinants in maternal health and well-being. Ministries with health as one of their responsibilities should work more closely with other sectors, such as those in charge of economic development and social protection. Strategies that favor the creation and reinforcement of social networks among women of reproductive age should be promoted. For example, group antenatal care could be tested in Rwanda because it has been shown to have positive effects on women’s satisfaction and in creating social support networks [55].

Finally, the quality of antenatal care in Rwanda should be assessed by not only evaluating practice based on the Rwandan antenatal guidelines but also benchmarking the guidelines based on the most recent evidence of effective interventions that should be included in antenatal practice.

## Abbreviations

ANC: Antenatal care
EQ-5D: European Quality of Life - 5 Dimensions
EQ-5D-3L: European Quality of Life - 5 Dimensions - 3 Levels
EQ-VAS: European Quality of Life - Visual Analogue Scale
HRQoL: Health-related quality of life
VAS: Visual analogue scale
